# A Novel Fibrin Matrix Derived from Platelet-Rich Plasma: Protocol and Characterization

**DOI:** 10.3390/ijms25074069

**Published:** 2024-04-06

**Authors:** Diego Delgado, Maider Beitia, Jon Mercader Ruiz, Pello Sánchez, Marta Montoya-Alzola, Nicolás Fiz, Mikel Sánchez

**Affiliations:** 1 Advanced Biological Therapy Unit, Hospital Vithas Vitoria, 01008 Vitoria-Gasteiz, Spain; diego.delgado@ucatrauma.com (D.D.); maider.beitia@ucatrauma.com (M.B.); jon.mercader@ucatrauma.com (J.M.R.); pello.sanchez@ucatrauma.com (P.S.); 2 Arthroscopic Surgery Unit, Hospital Vithas Vitoria, 01008 Vitoria-Gasteiz, Spain; marta.montoya@ucatrauma.com (M.M.-A.); nicolas.fiz@ucatrauma.com (N.F.)

**Keywords:** fibrin, scaffold, matrix, platelet-rich plasma, fibrinogen, platelets, growth factors

## Abstract

Although fibrin matrices derived from Platelet-Rich Plasma (PRP) are widely used in regenerative medicine, they have some limitations that can hinder their application. Modifying the composition of the PRP-derived fibrin matrix may improve its properties, making it suitable for certain medical uses. Three types of fibrin matrices were obtained: a PRP-derived fibrin matrix (FM), a PRP-derived fibrin matrix with a high fibrinogen content and platelets (FM-HFP) and a PRP-derived fibrin matrix with a high fibrinogen content (FM-HF). The fibrinogen levels, biomechanical properties and cell behavior were analyzed. The presence of platelets in the FM-HFP generated an inconsistent fibrin matrix that was discarded for the rest of the analysis. The fibrinogen levels in the FM-FH were higher than those in the FM (*p* < 0.0001), with a concentration factor of 6.86 ± 1.81. The values of clotting and swelling achieved using the FM-HF were higher (*p* < 0.0001), with less clot shrinkage (*p* < 0.0001). The FM had a significantly higher stiffness and turned out to be the most adherent composition (*p* = 0.027). In terms of cell viability, the FM-HF showed less cell proliferation but higher live/dead ratio values (*p* < 0.01). The increased fibrinogen and platelet removal in the FM-HF improved its adhesion and other biomechanical properties without affecting cell viability.

## 1. Introduction

Platelet-Rich Plasma (PRP) is a biological treatment based on obtaining, from a patient’s own blood, a volume of plasma with a concentration of platelets similar to or higher than the blood levels [1]. Its biosafety as an autologous product and its ease of production and application, as well as its promising results, have led to the rapid spread of research and numerous medical specialties in this area [2]. Another advantage is its versatility since it allows different types of formulations to be obtained. Thus, it is possible to obtain liquid formulations for injections and solid formulations consisting of fibrin clots/membranes [3]. The formation of these matrices is achieved via the activation of PRP, which triggers platelet degranulation and the coagulation cascade. This results in the interaction between thrombin and fibrinogen for the formation of a fibrin matrix (FM) [4]. PRP in its liquid form, as well as when formulated in a FM, presents therapeutic potential that makes it a valuable tool for various medical fields.

The mechanisms of action that favor these positive biological processes for healing are mainly mediated by biomolecules. They act on cell receptors and give rise to the resulting cellular response. Some of them are contained inside the α-granules of platelets, while others circulate in the plasma [5,6]. These factors are involved in the stimulation of cellular processes [1], in the early phases of tissue repair, in the coagulation system and in the immune response [7]. In these PRP-derived FMs, other types of proteins are found, such as thrombospondin-1 and lipoproteins, which participate in the processes of fibrinolysis and help delay the degradation of fibrin, allowing for a more sustained release of the various active molecules over time [8,9].

The ex vivo preparation of these fibrin scaffolds allows the application of PRP in medical processes where a liquid formulation is insufficient. For instance, its use can be applied in the treatment of wounds [10] or in surgical interventions addressing injuries of the musculoskeletal system [11]. As mentioned above, the placement of these scaffolds at the site of injury provides a controlled drug delivery system that stimulates tissue repair over time, extending the time of action of a conventional injection of a liquid PRP formulation [12,13].

However, the FMs derived from PRP have disadvantages that can limit their use on certain occasions. For example, the coagulation process through which FMs are formed takes a long time, making it unsuitable for applications requiring rapid matrix formation, such as in vivo processes [14]. In addition, the often-poor adhesion of these matrices, which can be compromised by their low fibrinogen concentration [15], also limits their application when prolonged adhesion of the clot to the tissue is required. Thus, commercial sealants based on these products vary in their proportions of thrombin and fibrinogen so as to modify product adhesion [16].

As for clot retraction, although it is beneficial in the biological processes of an organism [17], it can also be a limiting factor when using these matrices as scaffolds and fillers in surgical procedures [11,18]. According to previous studies, a higher concentration of fibrinogen in this type of matrix could improve its mechanical properties [19,20].

Accordingly, we hypothesize that modifying the composition of a PRP-derived FM may alter its mechanical characteristics, making it suitable for certain medical uses. Thus, the aim of this study was to develop a new protocol to obtain, from autologous PRP, a novel FM with a high fibrinogen content (FM-HF) whose biomechanical properties would be suitable for clinical application.

## 2. Results

### 2.1. Fibrinogen Concentration

**Figure 1:**
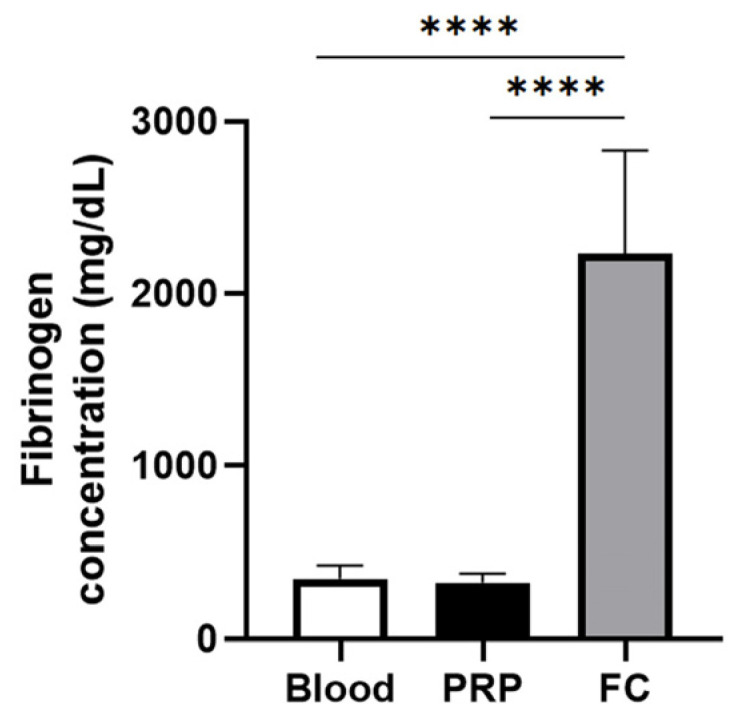
Fibrinogen concentration for matrix preparation. The graph represents the concentration of fibrinogen in blood (basal levels), in the PRP used to prepare the FM and in the fibrinogen concentrate (FC) used to create FM-HF. Error bars = standard deviation (*n* = 8). Statistically significant differences were calculated using one-way ANOVA (**** *p* < 0.0001).

Figure 1 depicts the fibrinogen levels of PRP and the fibrinogen concentrate used to create the FM and the FM-HF, respectively. While there is no difference in the fibrinogen concentration in PRP compared to that in the blood, the fibrinogen levels in the fibrinogen concentrate are significantly higher than those of both blood and PRP, reaching a concentration factor of 6.86 ± 1.81.

### 2.2. Clotting, Swelling and Retraction of the Matrices

Both matrices were formed within 2 to 3 min after mixing the components, with no time differences between them. However, the FM-HF underwent a gel state (Video S1) prior to final matrix formation that did not occur in the FM. The percentage of clotting achieved by the FM-HF was 82.85% ± 3.54, while that of the FM was significantly lower, at only 4.46% ± 1.55 (*p* < 0.0001).

In both matrices, the swelling ratio was low, with values below 1, and it was significantly higher for the FM-HF compared to the FM (0.87 ± 0.14 vs. 0.42 ± 0.12; *p* < 0.001).

Regarding retraction, the FM-HF decreased in its initial volume by only 4.91% ± 1.79, while the FM showed a much greater decrease (92.82% ± 2.17) (*p* < 0.0001). Table 1 shows all the data for each matrix.

**Figure 2:**
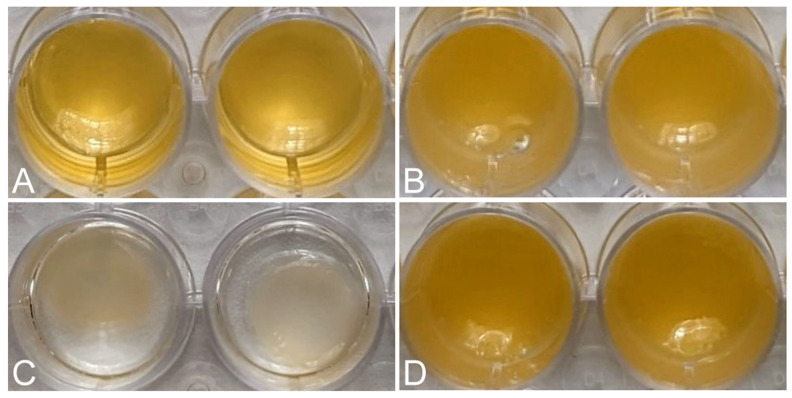
Retraction of the matrices. Macroscopic images show the initial volume of FM (**A**) and FM-HF (**B**) and the volume after 24 h, showing retraction in FM (**C**) and not in FM-HF (**D**).

Macroscopic images showed the reduction in volume of each matrix 24 h after its formation, due to the retraction of the matrices (Figure 2). The FM-HF formulations remained unchanged, while the FM clearly decreased in volume.

**Figure 3:**
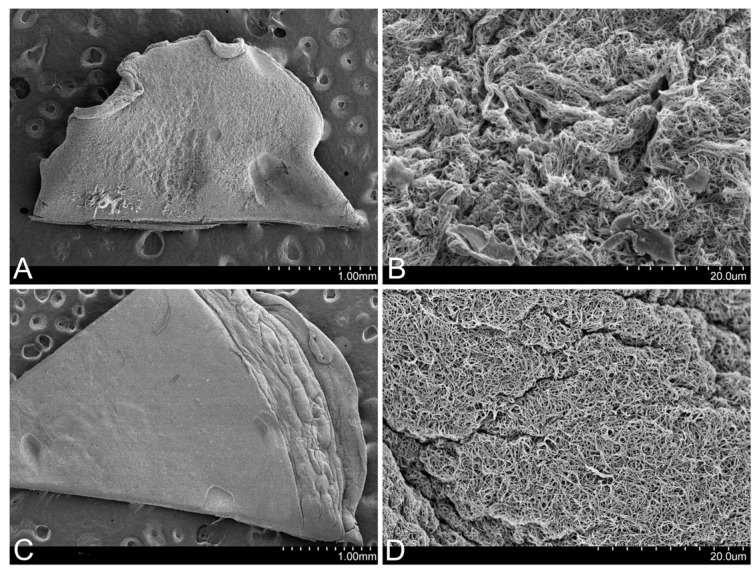
Surface of the matrices. SEM images show that the FM surface is rougher (**A**), with more contracted fibrin fibers (**B**). In contrast, the FM-HF surface is smoother (**C**), with more evenly arranged fibers (**D**).

In the images obtained using SEM, it could be observed that the FM presents a much more retracted and rougher surface than that of the FM-HF, on which the fibrin filaments are arranged in a more uniform manner (Figure 3).

### 2.3. Biomechanical Properties of the Matrices

**Figure 4:**
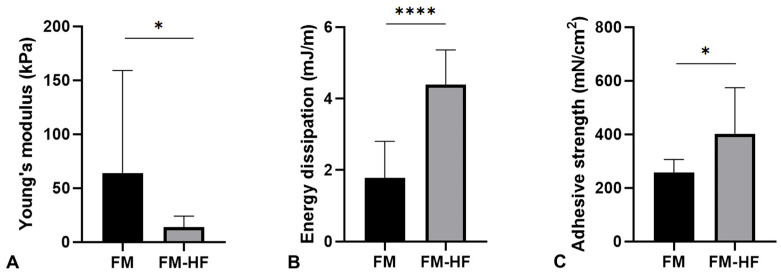
Mechanical properties of the matrices. The graphs represent Young’s modulus (stiffness) (**A**), the dissipation energy (cushioning) (**B**) and the adhesion capacity (**C**) of the FM and the FM-HF. Error bars = standard deviation (*n* = 8). Statistically significant differences were calculated using Student’s *t*-test (* *p* < 0.05; **** *p* < 0.0001).

The Young’s modulus data showed that the FM (64.16 kPa ± 95.23) had a significantly higher stiffness than the FM-HF formulation (13.89 kPa ± 10.18) (*p* = 0.023) (Figure 4A).

In terms of the energy dissipation, the FM-HF composite dissipated energy of 4.40 mJ/m, presenting a significantly higher cushioning capacity than the FM formulation, which dissipated energy of 1.78 mJ/m ± 1.02 (*p* < 0.0001) (Figure 4B).

Finally, the FM-HD turned out to be the most adherent composition, with an adhesion strength of 402.17 mN/cm^2^ ± 162.58, compared to the FM, which presented values of 256.92 mN/cm^2^ ± 46.71 (*p* = 0.027) (Figure 4C).

### 2.4. Cellular Viability of the Dermal Fibroblasts Seeded in the FM-HF and FM

**Figure 5:**
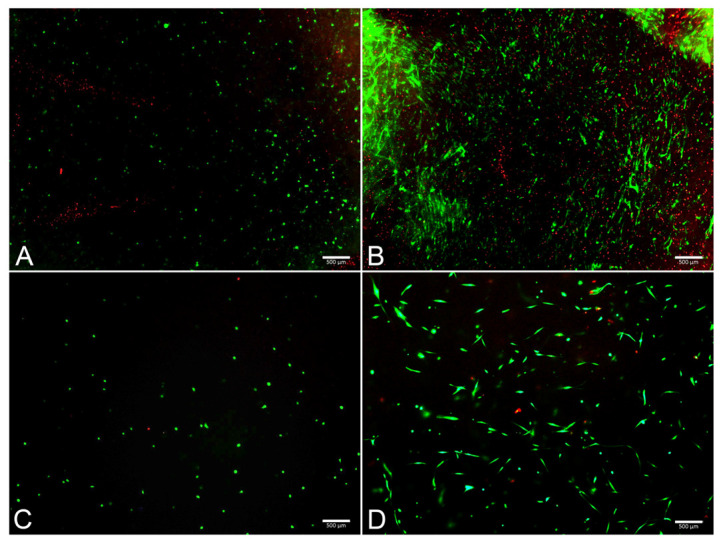
Images of live/dead assay. Fluorescence microscope images show live (green) and dead (red) cells at 24 h in the FM (**A**) and the FM-HF (**C**) and their evolution after 120 h of culture, with a greater increase in the number of live and dead cells in the FM (**B**) than in the FM-HF (**D**). Scale bar = 500 µm (5× objective).

**Figure 6:**
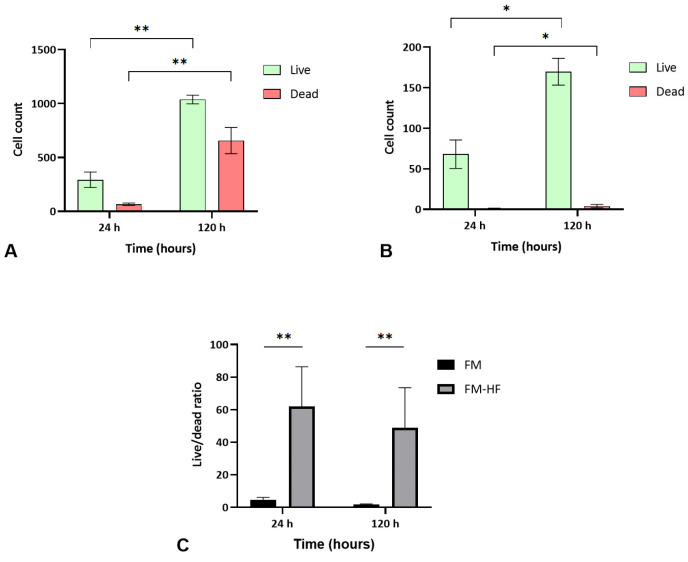
Cell viability. Data show a greater increase in both live and dead cells in the FM (**A**) than in the FM-HF (**B**). In both matrices, there is proliferation over time; however, the live/dead ratio is higher in the FM-HF (**C**). Error bars = standard deviation (*n* = 4). Statistically significant differences were calculated using Student’s *t*-test (* *p* < 0.05; ** *p* < 0.01).

The FM and FM-HF scaffolds were kept for 24 h and 120 h, and the live and dead cells were counted under a fluorescence microscope (Figure 5). The results showed a higher cell count in the FM scaffolds (*p* < 0.01) than in the FM-HF. In both cases, cells proliferated significantly from the 24 h to 120 h timepoint measurements, with faster growth in the FM (*p* < 0.01) than in the FM-HF (*p* < 0.05). However, higher dead cell levels were observed in the FM. In fact, when the live/dead ratio was considered, the FM-HF scaffold showed higher values than the FM (*p* < 0.01 and *p* < 0.01 at 24 h and 120 h, respectively) (Figure 6).

## 3. Discussion

The main findings of this work were improvements in the properties of a PRP-derived FM by removing platelets and increasing the levels of fibrinogen. The major advantages lay in the absence of shrinkage and retraction over time, a better consistency and cushioning capacity and greater adhesion strength, which allowed it to remain stable in the tissue. In addition, the toxicity of this type of biological matrix was not increased, which could compromise the cell viability of the target tissue.

Fibrin clot retraction is a physiological process within tissue repair. After clot formation, the reduction of its volume due to retraction favors aspects such as fibrin density, stiffness and stability [21]. However, in cases where FMs are prepared ex vivo or applied in vivo in order to be used for tissue repair during surgical procedures, this retraction may hinder the procedure. Therefore, avoiding clot retraction without losing the positive biomechanical properties that the clot entails could represent a breakthrough in the development of biological matrices for tissue repair. For this purpose, the development of the FM presented in this work was based on two main strategies, fibrinogen increase and platelet removal, but providing its biomolecular content in the scaffold.

This new fibrin matrix is characterized by its concentration of fibrinogen, as mentioned above, since the improvement of the FM’s mechanical properties is also related to fibrinogen levels [20,22]. Thus, in addition to using platelet-free plasma to create the FM-HF formulation, the fibrinogen levels were also increased. To achieve this, the platelet-free plasma fraction underwent a cryoprecipitation process using ethanol and low temperatures to promote the precipitation of fibrinogen, which was collected using centrifugation. This process was previously described by other authors, in which they used it to obtain an autologous fibrin gel [23] for successful use in thoracic [24] or maxillofacial surgery [25].

This increase in fibrinogen levels resulted in a new fibrin matrix that was much more consistent than a conventional one, as indicated by Young’s modulus, as well as higher energy dissipation, leading to better cushioning properties [26]. Finally, the increase in fibrinogen concentration also meant an increase in the adhesion of the FM-HF formulation. This fibrinogen-concentration-dependent adhesion has already been observed in previous studies [22,27,28]. In addition, the presence of platelets may also affect the adhesion of these matrices, as demonstrated by Irwin et al., who obtained better adhesion results with matrices derived from platelet-poor plasma than from PRP [29].

Regarding platelet deletion, these elements mediate the main mechanism of clot retraction [30], more specifically via the α2bβ3 receptor, which is highly expressed on the surface of platelets [31]. The different interactions and signaling pathways generated after the activation of this receptor lead to a shape change induced by the microtubule system, filopodia formation and clot retraction, mediated by actin reorganization [32]. In the present work, the high presence of platelets during processing caused platelet aggregation and retraction processes that prevented the final formation of a stable and homogenous fibrin matrix. This was finally achieved after the removal of platelets from the process. The resulting matrix (FM-HF) did not undergo a shrinkage process, maintaining its initial volume over time with a high percentage of clotting.

However, modifications that seek to mechanically enhance fibrin matrices may adversely affect their biological properties. The removal of platelets could also be a limiting factor for this type of autologous matrix, as these elements contain a large number of molecules involved in biological processes that stimulate tissue repair [33]. It is reasonable to think that the removal of platelets might diminish their ability to stimulate tissue repair. For this reason, the method described in this work to elaborate the FM-HF formulation includes the use of a fraction of PRP. Following its activation, the platelets release their content [34] and thus obtain a serum with thrombin and growth factors. Combining this serum with the fibrinogen concentration generates the fibrin clot, and the molecules released by the platelets present in the serum fraction anchor in the fibrin mesh through the heparin sulfate domains [35]. This results in a scaffold without platelets but with their platelet content and the rest of the plasmatic biomolecules [6]. Fibrin degradation over time would allow a gradual and controlled release of fibrin-anchored growth factors. The kinetics of this growth factor delivery seem to consist of an initial rapid release of 30% of the bioactive content after 1 h of incubation and a steady-state release of almost 70%. The autologous fibrin matrix retained almost 30% of the amount of the growth factors after 8 days of incubation [36]. However, in the case of the FM-HF, this release would be delayed due to slower degradation because of its high fibrinogen content.

Increased fibrinogen levels can also lead to alterations in the biological properties of scaffolds. The swelling ratio of the scaffolds is a factor that may condition the relationship between the matrices and the cells. In both formulations, these values were low, although they were higher in the FM-HF scaffold, suggesting that its nutrient diffusion capacity would not be compromised and consequently nor would the cell viability [37]. Moreover, the modification of the cushioning properties, in addition to being a mechanical improvement, can also favor other biological processes. Indeed, Abdel-Sayed et al. showed that scaffolds with higher levels of energy dissipation had better chondrogenic properties [38], making the use of such matrices highly suitable in chondral defects [39].

In contrast, previous studies have shown that increased fibrinogen can affect other cellular processes such as cell migration or proliferation. This phenomenon was described in different cell populations, such as retinal pigment epithelial cells [40], neutrophils [41] and mesenchymal stem cells [42,43,44]. The results obtained in the present work are in line with these studies, as the cell proliferation was lower in the FM-HF formulation than in the FM. The increase in fibrinogen concentration could lead to a reduced capacity for cellular interactions and the diffusion of nutrients and other biomolecules, resulting in a decrease or even inhibition of cell proliferation [40]. In the present work, this growth was not inhibited but progressed slowly over time, possibly because fibrinogen did not reach excessive concentrations, as in commercial products [29]. On the other hand, the cell proliferation values in the FM formulation, which had a lower concentration of fibrinogen, were much higher. This cell growth occurred in a short period of time, although it was accompanied by an increase in cell death. This higher mortality may be due precisely to the high rate of proliferation in a scaffold that was rapidly shrinking in size, thus depleting the nutrients and space that supported the cells [45]. These levels of cell death were not observed in the FM-HF scaffold, indicating an absence of cellular toxicity, as well as slower and more sustained cell growth over time.

Overall, this work describes the process required to create a new PRP-derived fibrin matrix which, thanks to its improved biomechanical properties, facilitates improved application in the medical–surgical field. Furthermore, fast polymerization allows for easy application in liquid form to the target tissue, where a highly adherent and stable scaffold will be generated and maintained over time. These properties make the new fibrin matrix suitable for applications in arthroscopic surgery. The administration in a liquid state and subsequent polymerization in the target tissue would allow its application to this type of intervention. In addition, its adhesion and lack of shrinkage would also make it suitable for use in the treatment of wounds or other types of tissue defects.

This study has a number of limitations. First, commercial products already exist that may facilitate clinical use, even if they do not have the biological advantages of autologous products. The use of these commercial products as controls would have provided more information for the study, though the characterization of these products already exists in the scientific literature (Table S1) [23,46,47]. Second, only the cell viability was measured for the analysis of the biological properties; in further studies, cell assays of inflammation or cell migration should be considered in the future. Finally, in vivo and clinical studies are needed to evaluate aspects such as new tissue formation, scar generation, safety and effectiveness. However, these results will pave the way for new approaches and studies for the creation of PRP-derived autologous scaffolds. Future studies could focus on translational and clinical research addressing the use of this matrix for the treatment of different pathological conditions, such as wounds or osteochondral defects.

## 4. Materials and Methods

### 4.1. Preparation of the Formulations

For the preparation of the formulations in the study, whole blood was withdrawn from healthy donors into tubes of 9 mL containing 3.8% (*w*/*v*) sodium citrate (9 NC coagulation sodium citrate 3.8% tube, Greiner Bio-One, Kremsmünster, Austria). Ethical approval was obtained from the Ethics Committee of Araba University Hospital (HUA) (2015-012, 27 March 2015), and written consent was obtained from the patients. Two plasma fractions were obtained from the blood, which provided the main components for making the fibrin matrices: thrombin and fibrinogen. Following the blood collection, different protocols were written up for obtaining each type of fibrin matrix (Figure S1).

#### 4.1.1. PRP-Derived Fibrin Matrix (FM)

After the blood collection, we centrifugated four tubes at 580× *g* for 8 min at room temperature in order to obtain the PRP fractions (BTI Biotechnology Institute, Vitoria-Gasteiz, Spain). The resulting fractions in each tube were divided into 2 halves: an upper-half fraction with fewer platelets and a lower-half fraction with a platelet concentration between 1.5 and 2.5 times higher compared to that of the peripheral blood. We collected the upper fractions in a 9 mL tube and the lower fractions in another 9 mL tube. (Z No Additive tube, Greiner Bio-One, Kremsmünster, Austria).

We added calcium chloride (10% *w*/*v*) to the tube containing the upper fraction in order to activate the platelets and trigger the coagulation process. Once a clot was formed, next came its retraction and the exudation of serum containing growth factors and thrombin.

We added this serum to the tube with the lower fraction of PRP (*v*/*v*: 1/1). When the two components were mixed, there occurred an interaction between the thrombin in the serum and the fibrinogen in the PRP, forming the final fibrin matrix.

Although in standard practice this fibrin matrix is made with the addition of initial calcium chloride, the method used in this study is intended to be compatible and comparable with the new protocol.

#### 4.1.2. The PRP-Derived Fibrin Matrix with a High Fibrinogen Content and Platelets (FM-HFP)

Ten tubes of the collected blood were centrifuged at 580× *g* for 8 min at room temperature in order to obtain the PRP fraction (BTI Biotechnology Institute, Vitoria-Gasteiz, Spain). We collected all the PRP whole columns in five 9 mL tubes (Z No Additive tube, Greiner Bio-One, Kremsmünster, Austria). We used one PRP tube to obtain the serum containing growth factors and thrombin. For this purpose, we added calcium chloride to the tube (10% *w*/*v*), causing clot formation and the subsequent retraction and exudation of the serum.

We used the other four PRP tubes to obtain the fibrinogen and platelets. For this, we added ethanol 96% (PanReac AppliChem, Barcelona, Spain) to the PRP tubes (10% *v*/*v*), and after mixing them gently, they were incubated at 4 °C for 30 min.

Next, we centrifuged these tubes at 580× *g* for 8 min at room temperature in order to precipitate the platelets and fibrinogen and after centrifugation. The supernatant was discarded from each tube, keeping the pellets formed. We incubated the pellets at 37 °C until dissolution and collected them in the same tube, giving a final volume of the fibrinogen and platelet concentrate of 1.5–2 mL.

To create the final fibrin matrix, we mixed the fibrinogen and platelet concentrate with the serum. The serum contained both thrombin and growth factors. We used equal volumes of each component.

However, during the creation of this fibrin matrix, the platelet and fibrinogen concentrate presented a heterogeneous appearance due to the formation of small fibers and clots, resulting in an inconsistent and heterogenous fibrin matrix which was discarded for the rest of the analysis (Figure S2A,B).

#### 4.1.3. The PRP-Derived Fibrin Matrix with a High Fibrinogen Content (FM-HF)

Ten tubes of the collected blood (9 NC coagulation sodium citrate 3.8% tube, Greiner Bio-One, Kremsmünster, Austria) were used for this protocol. We used two of them to obtain the serum containing platelet and plasma biomolecules and thrombin. For this purpose, we centrifuged these two tubes at 580× *g* for 8 min at room temperature in order to obtain the PRP fractions. These two PRP whole columns were collected in one 9 mL tube (Z No Additive tube, Greiner Bio-One, Kremsmünster, Austria). As before, we added calcium chloride to the tube (10% *w*/*v*), causing clot formation and the subsequent retraction and exudation of the serum.

We used the other eight blood tubes to obtain the fibrinogen concentrate without platelets. We centrifuged these tubes at 1500× *g* for 15 min at room temperature in order to achieve a platelet-free plasma fraction. We collected all these plasma columns in four 9 mL tubes (Z No Additive tube, Greiner Bio-One, Kremsmünster, Austria) and mixed them gently with ethanol 96%, pharma-grade (PanReac AppliChem, Spain) (10% *v*/*v*). We incubated the tubes at 4 °C for 30 min.

The tubes were centrifuged at 580× *g* for 8 min at room temperature to precipitate the fibrinogen. The supernatant was discarded from each tube, keeping the fibrinogen pellets, and we incubated them at 37 °C until dissolution. We collected the dissolved pellets in the same tube, reaching a final volume of the fibrinogen concentrate of 1.5–2 mL.

To create the final fibrin matrix, we mixed the fibrinogen concentrate with the serum. The serum contained both thrombin and growth factors. We used equal volumes of each component.

Unlike the fibrinogen with platelets, all the initial components and the final matrix were homogeneous and consistent, so it was this fibrin matrix that was selected for the various different analyses. (Figure S2C,D).

### 4.2. Fibrinogen Level Measurements

We measured the fibrinogen levels using a coagulation analyzer (STA Compact Max, Stago, Asnières-sur-Seine, France). We compared the fibrinogen levels present in the blood plasma, in the PRP and in the fibrinogen concentrate samples.

### 4.3. Clotting, Swelling and Retraction

We compared the clotting, swelling and retraction properties between the FM and FM-HF. In order to prepare the formulations, we mixed 300 µL of thrombin serum and 300 µL of PRP for the FM, and we used 300 µL of thrombin serum and 300 µL of fibrinogen for the FM-HF.

We measured the clotting time for each formulation from the moment the two components were mixed until clot formation. The clotting or coagulation percentage [(W_f_/W_i_) × 100] was analyzed, taking into account the initial weight of the mixture (W_i_) with the weight of the matrix 24 h after its formation (W_f_).

The swelling ratios were determined using a gravimetric method. We calculated the swelling ratio as [(W_w_ − W_d_)/W_d_], where W_w_ is the weight of the wet matrix and W_d_ is its weight 24 h after forming and drying. After weighing the dry scaffolds, we plunged them into PBS for 24 h. Thereafter, we weighed the wet scaffolds again and calculated the swelling ratio. Immediately before weighing, any excess surface water was carefully removed.

Regarding retraction, we calculated the volume of the matrices at their formation, as well as 24 h later. We also took macroscopic photographs immediately after matrix formation, as well as after 24 h. In addition, we used scanning electron microscopy (SEM) to take microscopic images 24 h after the matrix formation to evaluate its structure. We rinsed the formulations with phosphate-buffered saline, fixed them using 2% glutaraldehyde in 0.1 M cacodylate buffer for 4 h and washed them in cacodylate–sucrose buffer. Then, we post-fixed the samples with osmium tetroxide for 1 h, washed them again and dehydrated them with serial concentrations of ethanol. The samples were critical-point-dried (tousimis Autosamdri 814; Tousimis, Rockville, MD, USA), sputter-coated with 5 nm gold (Edwards E306A; Edwards Vacuum, Burgess Hill, UK)) and subsequently examined under a scanning electron microscope (Hitachi S-4800; Hitachi, Tokyo, Japan).

### 4.4. Mechanical Tests

To determine Young’s modulus and the dissipated energy of the FM and FM-HF, we carried out instrumented indentation using a spherical indenter. In these tests, a calibrated spherical indenter with a diameter of 5 mm was pressed onto the sample to be studied, while recording the load used to penetrate the indenter, as well as the penetration distance at each moment. The indentations were performed directly on 24-well plates, where the different formulations were elaborated (Figure S3A). The sphere penetrated the sample at a constant speed of 50 μm/s until it reached a depth equivalent to 20% of the initial thickness of the sample. Once this penetration was reached, the direction of movement was reversed, unloading the indenter at a constant speed of 100 μm/s.

To determine the adhesiveness of both types of matrices, we covered two cylinders with a gauze, each impregnated with the formulation to be studied in its liquid state, and the two gauzes were then pressed together. After waiting the necessary time for the formation of the matrices, we made an attempt to detach the cylinders by separating the two glued gauzes (Figure S3B). We calculated the resulting adhesion strength by identifying the area of the glued gauzes and the maximum force required for their separation during the test.

A zwickiLine Z1.0 uniaxial testing machine (ZwickRoell, Ulm, Germany) was used for these tests. The load cell was a ZwickRoell Xforce P with a maximum load of 50 N.

### 4.5. Cell Assays

Normal Human Dermal Fibroblasts (NHDFs) (Lonza; Basel, Switzerland) were cultured using Fibroblast Basal Medium (FBM) (Lonza; Basel, Switzerland) at 37 °C and 5% CO_2_.

When the cells reached confluence, they were trypsinized and seeded in the scaffold precursor mix so that they stayed embedded into it after coagulation. In the case of the fibrinogen, 10^3^ cells were seeded per well. Considering that the FM underwent greater retraction, reaching a final volume of 12% compared to the FM-HF, 120 cells were seeded per well to ensure a similar confluency. We incubated the FM and FM-HF scaffolds for 24 and 120 h, respectively. Subsequently, we quantified the number of live and dead cells using fluorescence microscopy. Four replicates were considered for each scaffold so that each one could be measured at a specific timepoint.

A LIVE/DEAD kit (Thermo Fisher Scientific, Waltham, MA, USA) was employed for measurement of the live and dead cells following the manufacturer’s instructions. The fluorescence emitted by both calcein and ethidium was measured under a fluorescence microscope, and each cell was manually counted.

### 4.6. Statistical Analysis

The distribution of the samples was assessed using Shapiro–Wilk’s normality test. The different variables were determined by the mean and the standard deviation of the parametric data. Comparisons were performed using ANOVA and Student’s *t*-test. Data were considered statistically significant when *p* < 0.05. GraphPad Prism^®^ software version 9.5 (San Diego, CA, USA) was used for the statistical analysis.

## 5. Conclusions

The removal of platelets and the increase in the fibrinogen levels result in the rapid formation of a fibrin matrix with a high capacity for adhesion, consistency and cushioning and no shrinkage, which favors its maintenance over time. Moreover, the improvement of these mechanical properties does not lead to an increase in cell mortality or toxicity but results in slow and sustained cell growth.

## Figures and Tables

**Table 1:** Clotting, swelling and retraction of fibrin matrices.

	FM	FM-HF	*p* Value
W_i_ (g) (mean ± SD)	0.60 ± 0.01	0.60 ± 0.02	0.742
W_f_ (g) (mean ± SD)	0.03 ± 0.01	0.50 ± 0.03	<0.0001 ****
Clotting (%) (mean ± SD)	4.46 ± 1.55	82.85 ± 3,54	<0.0001 ****
W_d_ (g) (mean ± SD)	0.01 ± 0.00	0.07 ± 0.00	<0.0001 ****
W_w_ (g) (mean ± SD)	0.02 ± 0.00	0.13 ± 0.00	<0.0001 ****
Swelling ratio	0.42 ± 0.12	0.87 ± 0.14	<0.001 ***
V_i_ (mm^3^) (mean ± SD)	102.74 ± 5.23	103.71 ± 4.29	0.756
V_f_ (mm^3^) (mean ± SD)	7.35 ± 2.10	100.98 ± 4.91	<0.0001 ****
Retraction (%) (mean ± SD)	92.82 ± 2.17	4.91 ± 1.79	<0.0001 ****

FM: Fibrin matrix; FM-HF: fibrin matrix with high fibrinogen content; W_i_: initial weight; W_f_: weight after 24 h; W_d_: weight after drying; W_w_: weight after rehydration; V_i_: initial volume; V_f_: volume after 24 h. (*** *p* < 0.001; **** *p* < 0.0001).

## Data Availability

The data presented in this study are available within the article. Additional inquiries may be sent to the corresponding authors.

## Supplementary Materials

The following supporting information can be downloaded at https://www.mdpi.com/article/10.3390/ijms25074069/s1.
