# Supplementary material for: A Novel Fibrin Matrix Derived from Platelet-Rich Plasma: Protocol and Characterization
Source: Int J Mol Sci. 2024 Apr 6;25(7):4069. doi: 10.3390/ijms25074069 (PMC11012499; doi:10.3390/ijms25074069)
Supplement: Supplementary file 1 [file ijms-25-04069-s001.zip › Table S1_R1.pdf]

Table S1. Comparison between autologous and commercial matrices

	FM	FM-HF	Tisseel®
Fibrinogen Concentration	+	++	+++ [23]
Young's modulus	+++	+	+ [46]
Adhesiveness	+	+++	+++ [23]
Cell proliferation	+++	++	+ [47]

FM: Fibrin Matrix; FM-HF: fibrin matrix with high fibrinogen content; the degree of effect is classified as low (+), medium (++) or high (+++), comparing the 3 formulations with each other.
