# Supplementary figures and images for: A Novel Fibrin Matrix Derived from Platelet-Rich Plasma: Protocol and Characterization
Source: Int J Mol Sci. 2024 Apr 6;25(7):4069. doi: 10.3390/ijms25074069 (PMC11012499; doi:10.3390/ijms25074069)

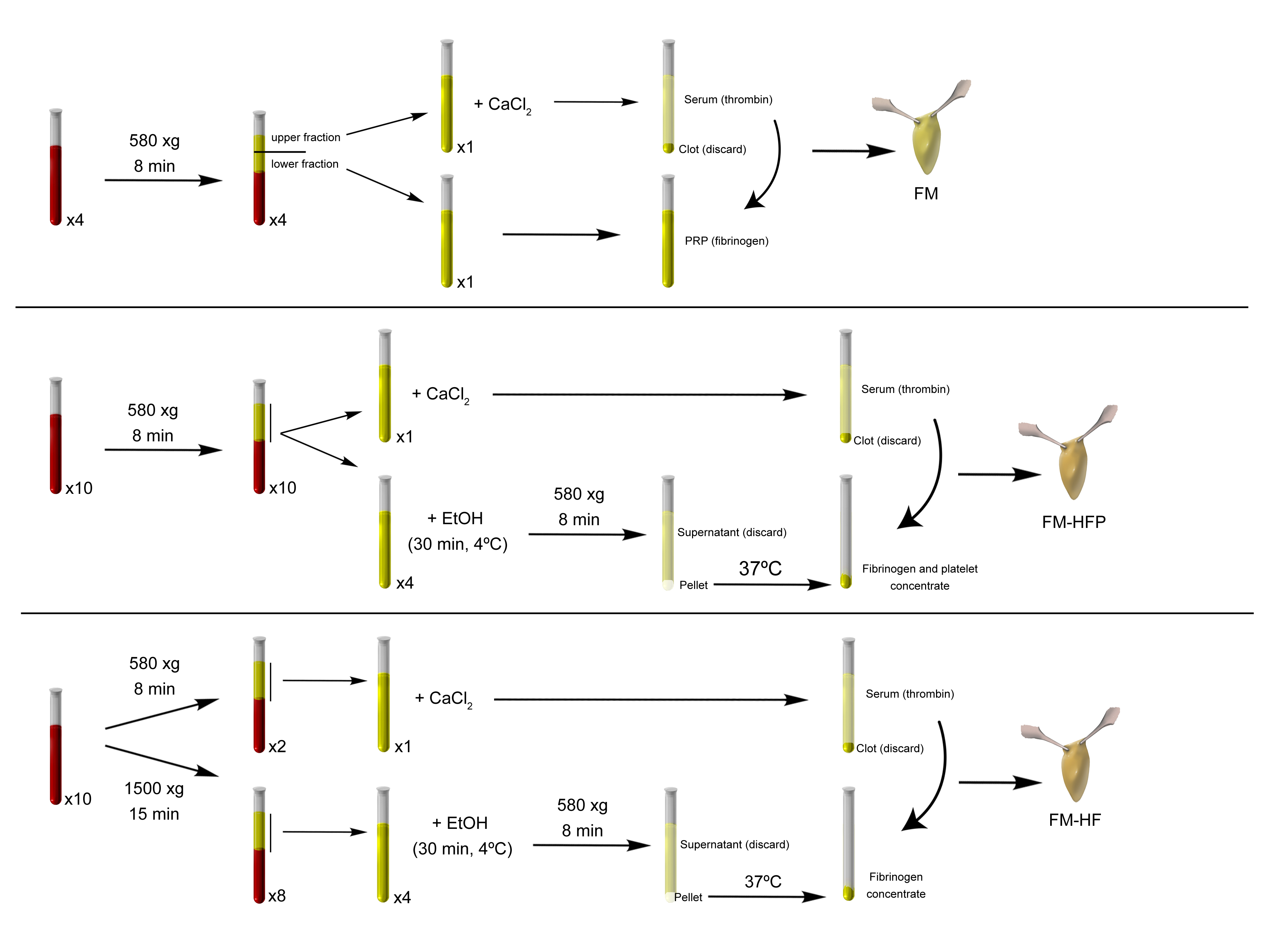

Supplement: Supplementary file 1 [file ijms-25-04069-s001.zip › Figure S1.jpg]

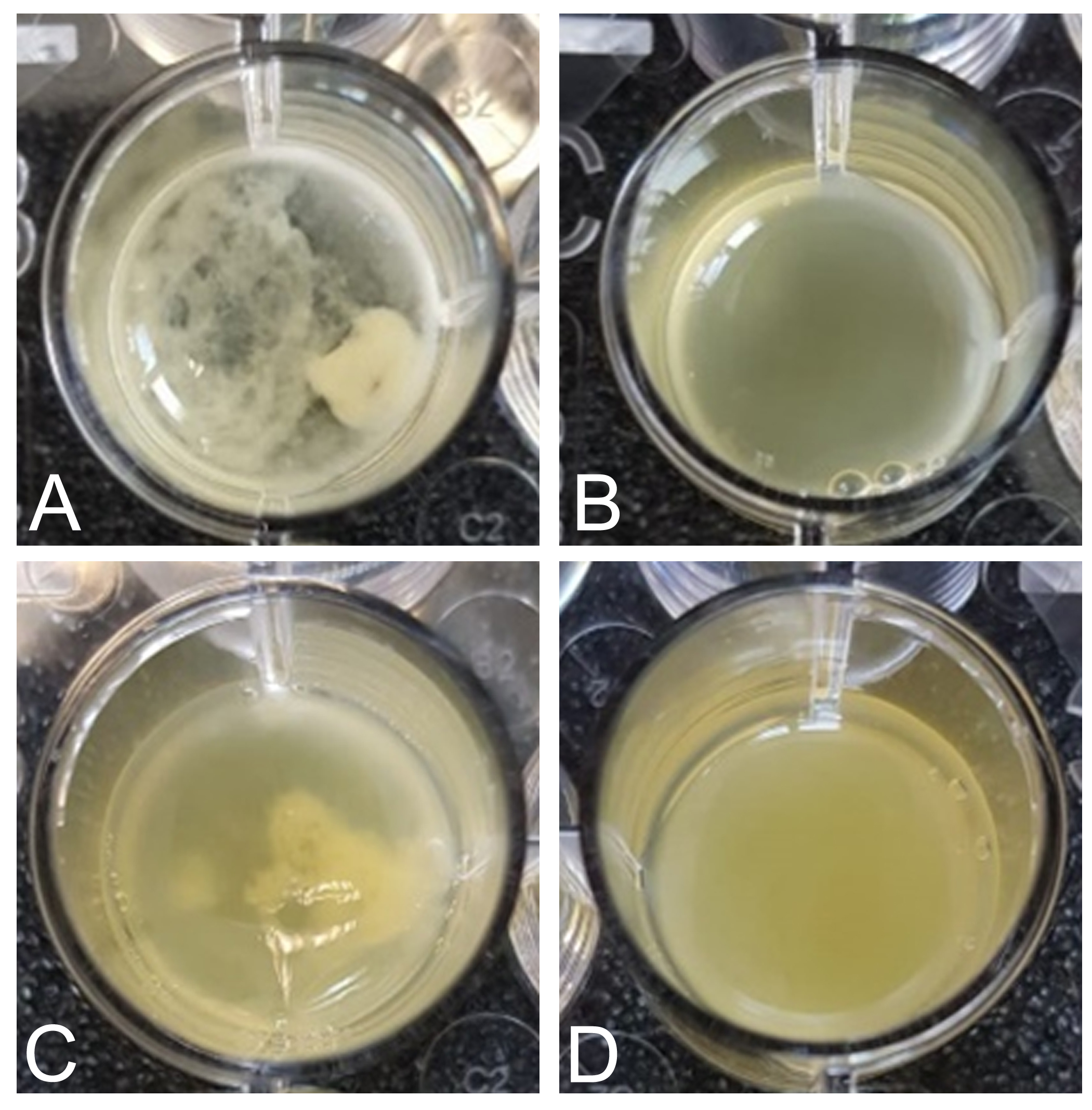

Supplement: Supplementary file 1 [file ijms-25-04069-s001.zip › Figure S2.jpg]

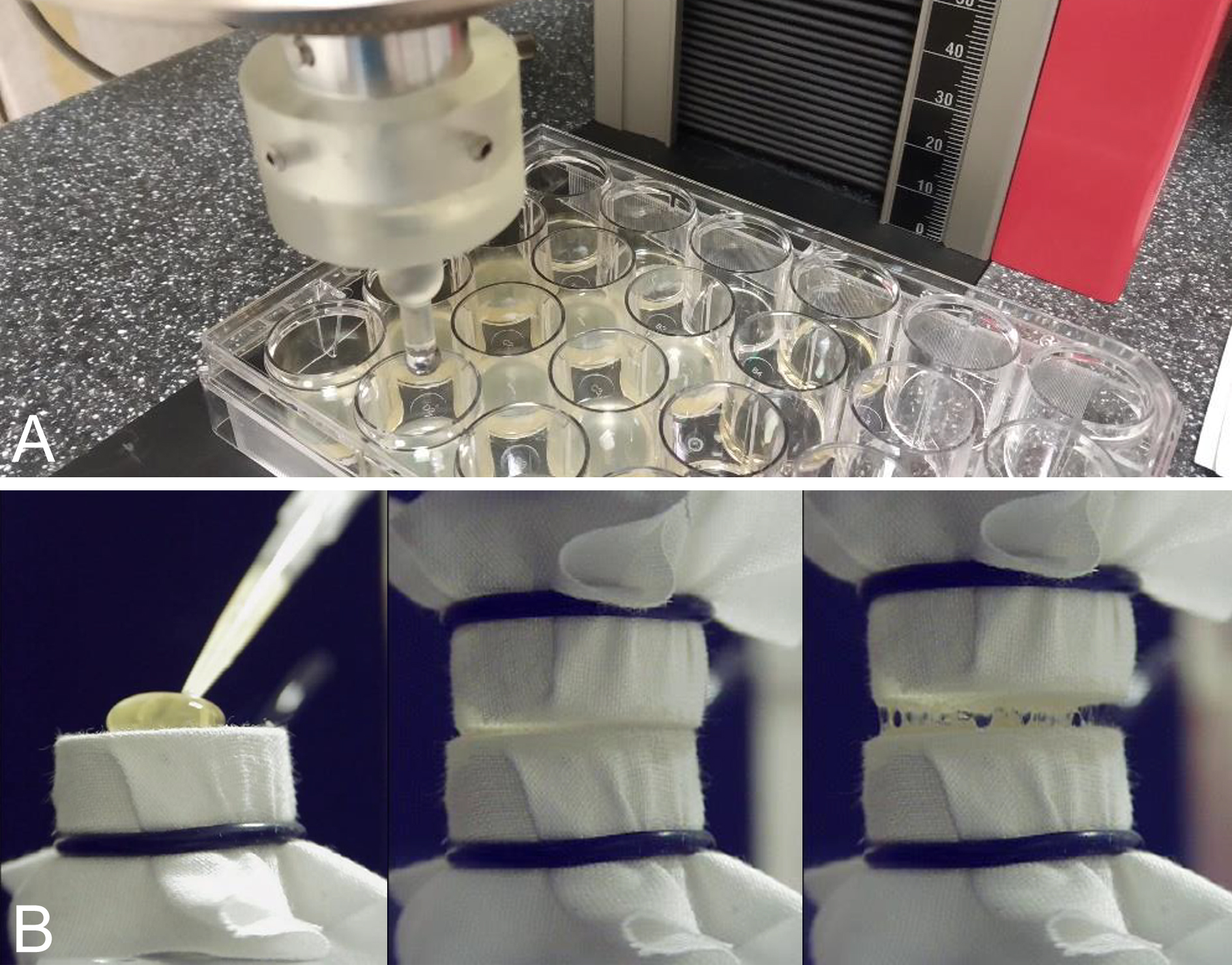

Supplement: Supplementary file 1 [file ijms-25-04069-s001.zip › Figure S3.jpg]
